# Dosimetric evaluation of multilumen intracavitary balloon applicator rotation in high‐dose‐rate brachytherapy for breast cancer

**DOI:** 10.1120/jacmp.v15i1.4429

**Published:** 2014-01-06

**Authors:** Yongbok Kim, Mark G. Trombetta

**Affiliations:** ^1^ Department of Radiation Oncology University of Arizona Tucson AZ; ^2^ Department of Radiation Oncology Allegheny General Hospital Pittsburgh PA; ^3^ Temple University School of Medicine Allegheny Campus Pittsburgh PA USA

**Keywords:** dosimetric evaluation, multilumen intracavitary balloon applicator, applicator rotation, high‐dose‐rate brachytherapy, breast cancer

## Abstract

The objective of this work is to evaluate dosimetric impact of multilumen balloon applicator rotation in high‐dose‐rate (HDR) brachytherapy for breast cancer. Highly asymmetrical dose distribution was generated for patients A and B, depending upon applicator proximity to skin and rib. Both skin and rib spacing was ≤0.7 cm for A; only rib spacing was ≤0.7 cm for B. Thirty‐five rotation scenarios were simulated for each patient by rotating outer lumens every 10° over $\pm 180^{\circ }$ range with respect to central lumen using mathematically calculated rotational matrix. Thirty‐five rotated plans were compared with three plans: 1) original multidwell multilumen (MDML) plan, 2) multidwell single‐lumen (MDSL) plan, and 3) singledwell single‐lumen (SDSL) plan. For plan comparison, planning target volume for evaluation (PTV_EVAL) coverage (dose to 95% and 90% volume of PTV_EVAL) (D95 and D90), skin and rib maximal dose (Dmax), and normal breast tissue volume receiving 150% (V150) and 200% (V200) of prescribed dose (PD) were evaluated. Dose variation due to device rotation ranged from −5.6% to 0.8% (A) and −6.5% to 0.2% (B) for PTV_EVAL D95; −5.2% to 0.4% (A) and −4.1% to 0.7% (B) for PTV_EVAL D90; −2.0 to 18.4% (A) and −7.8 to 17.5% (B) for skin Dmax; −11.1 to 22.8% (A) and −4.7 to 55.1% (B) of PD for rib Dmax, respectively. Normal breast tissue V150 and V200 variation was <1.0 cc, except for −0.1 to 2.5 cc (B) of V200. Furthermore, 30° device rotation increased rib Dmax over 145% of PD: 152.9% (A) by clockwise 30° rotation and 152.5% (B) by counterclockwise 30° rotation. For a highly asymmetric dose distribution, device rotation can outweigh the potential benefit of improved dose shaping capability afforded by multilumen and make dosimetric data worse than single‐lumen plans unless it is properly corrected.

PACS number: 87.53.Jw

## I. INTRODUCTION

As a breast conservation therapy following lumpectomy, whole‐breast irradiation (WBI) has been considered as a standard of care with comparable disease free and overall survival to mastectomy for early stage of breast cancer.^1^, ^2^ The irradiated volume of breast tissue in WBI technique is larger and overall treatment time is longer (five to seven weeks vs. one to two weeks) than accelerated partial‐breast irradiation (APBI) technique. The effectiveness of APBI has been investigated in comparison with WBI under a joint clinical trial by the National Surgical

Adjuvant Breast and Bowel Project and the Radiation Therapy Oncology Group (NSABP B‐39/RTOG 0413).^(3)^ High‐dose‐rate (HDR) interstitial brachytherapy with multiple catheters was first adapted as an APBI, but it often requires the complexity of implantation (up to approximately 20 catheters) to encompass the lumpectomy cavity with an appropriate margin.^(4)^ To deliver a single fraction of radiation to the tumor bed, intraoperative radiotherapy technique has been used as an APBI.^(5)^ As a noninvasive procedure, three‐dimensional conformal radiation therapy (3D CRT) technique is attractive, but requires a large margin to account for uncertainty in patient setup and breast/organ motion. A recent interim randomized clinical trial data reveal that APBI using 3D CRT significantly increases adverse cosmesis and late radiation toxicity compared to WBI.^(6)^

The advantage from the use of a single central lumen intracavitary balloon applicator such as MammoSite (Hologic Inc., Bedford, MA) in HDR brachytherapy is the simplicity and reproducibility over the course of radiation treatment. Therefore, it has been a popular option as an APBI following lumpectomy for early‐stage breast cancer patients.^(7)^ However, in a specific case where the single‐lumen MammoSite balloon is located proximally to organs at risk (OARs) such as skin and rib, the benefit from the single‐lumen MammoSite balloon has to be reconsidered for achieving clinically adequate target coverage while simultaneously keeping the dose to OARs as low as possible. Because the available dwell positions are limited to the single central lumen, nearly spherically shaped symmetric dose distribution is generated. Hence, the dose to OARs cannot be reduced without compromising target coverage in many cases.^8^, ^9^, ^10^, ^11^ In order to reduce skin dose, Edmundson et al.^(7)^ suggested a simple technique which takes advantage of the source anisotropy by placing applicator axis normal to the skin. With this approach the maximal skin dose of 35 Gy was reduced by 10 Gy. They also proposed a modified surgical procedure using a vertical incision to increase balloon‐to‐skin distance. Therefore, the NSABP B‐39/RTOG 0413 phase III trial protocol^(3)^ requires minimum balloon‐to‐skin distance of ≥0.7 cm for singlelumen MammoSite technique. The protocol allows 0.5∼0.7 cm of minimum balloon‐to‐skin distance if the maximal dose (Dmax) to skin is ≤145% of prescribed. There is no specific dose limit to rib. A treatment plan requires that more than 90% of the prescribed dose covers at least 90% of target volume in order to be clinically acceptable.

To produce an asymmetric dose distribution, the Contura multilumen balloon (MLB) (Bard Biopsy Systems, Tempe, AZ) has been developed and used in the clinic. The Contura MLB applicator introduced four additional lumens with a 0.5 cm offset from the single central lumen. By optimizing dwell‐time distribution for all possible dwell positions identified by five lumens, a treatment plan can achieve the dosimetric goal proposed by the Contura registry study.^9^, ^10^, ^11^ For target coverage, ≥95% of the prescribed dose should cover ≥95% of target volume (D95≥95%). Skin and rib Dmax are required to be ≤125% and 145% of the prescribed dose, respectively. In addition, the volume of normal breast tissue receiving 150% (V150) and 200% (V200) of the prescribed dose is limited to 50 cc and 10 cc, respectively. Due to the potential dose shaping capability resulting from four outer lumens, the protocol allowed as short as 0.3 cm of minimum balloon‐to‐skin distance. The dosimetric advantage of the Contura applicator over the MammoSite single‐lumen applicator has previously been reported.^9^, ^10^, ^11^

In order to properly translate the dosimetric benefit from the Contura applicator into clinically improved patient outcomes, verification of reproducibility of asymmetric dose distribution (device rotation) is necessary prior to each treatment. Ouhib et al.^(12)^ confirmed that the use of device alignment with an external skin mark is an accurate and reliable method to verify applicator rotation. In the routine clinical practice, device rotation is always checked prior to each fraction. However, to our knowledge there is no reported study or guideline available to investigate the specific dosimetric impact due to device rotation. Because each clinical case has its own asymmetric dose distribution, the dosimetric impact from device rotation may be patient‐specific, depending upon the geometry and proximity of the device relative to neighboring OARs. This institutional review board‐approved study evaluated the dosimetric impact of Contura MLB rotation for two representative clinical cases.

## II. MATERIALS AND METHODS

### A. Treatment planning and verification prior to delivery

**Figure 1:**
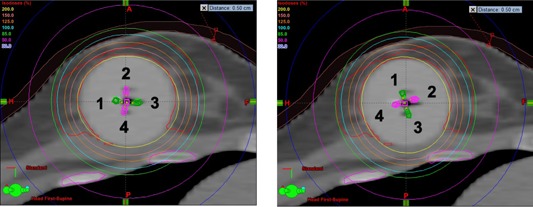
Comparison of isodose distribution between the original MDML plan (a) and a rotated plan (b) with 70° clockwise rotation for Case A. The isodose lines in (a) demonstrate an asymmetric dose distribution conformal to PTV_EVAL (red contour), while those in (b) show the unintended dose increase to skin and rib with less conformal dose to PTV_EVAL. The five multilumen catheters and their rotation are easily visualized in these two‐dimensional (2D) images at the central section of the balloon. In these 2D images, balloon‐to‐skin distance is larger than 0.5 cm. Note that the minimum balloon‐to‐skin distance can be accurately measured by a comprehensive review of 3D balloon relative to the skin in 3D planning CT images. For Case A, it was 0.5 cm and located in the distal (not central) section of balloon.

A fractional dose of 3.4 Gy was delivered with twice daily fractions at least 6 hours apart over five consecutive working days using an HDR ${}^{192}\text{Ir}$ source (total of 34 Gy). Computed tomography (CT) images with 2 mm slice thickness were used for treatment planning by following Contura registry study guidelines. The planning target volume for evaluation (PTV_EVAL) (red contour in Fig. 1) was created as a uniform 1 cm expansion from the balloon surface, excluding breast tissue 0.5 cm from the skin surface and excluding chest wall and pectoralis muscle. All contours were segmented by a physicist and reviewed by a physician. A multiple‐dwell position method was used together with a volume optimization technique^13^, ^14^ commercially available in a treatment planning system (TPS) (BrachyVision version 8.1.2.0, Varian Medical Systems Inc., Palo Alto, CA). Dwell‐time distributions corresponding to possible dwell positions were optimized to satisfy the Contura dosimetric goals. This commercial TPS does not take tissue inhomogeneity into account in the dose calculation. In the dwell‐time optimization procedure, target coverage has the highest priority (i.e., penalty value of 100) with modest constraint (i.e., 50) on OARs dose limit. If dose to OARs is clinically high or unacceptable (i.e., violating the Contura registry study), the constraint on the target is relaxed (i.e., 80) and hard constraint (i.e., 100) is set on OARs dose limit in the inverse planning optimization. A set of dose constraints on target and OARs is modified and dosimetry data of the optimized plan are evaluated accordingly until a clinically desirable plan is obtained. If these parameters cannot be met, a treatment plan should at least meet the dosimetry guidelines of the NSABP B‐39/RTOG 0413 protocol. Moreover, to demonstrate the superiority of multidwell position for multilumen (MDML) plan, the Contura protocol requires two additional single‐lumen plans: single‐dwell (SD) position for single central lumen (SDSL) plan and multidwell position for single central lumen (MDSL) plan.

There are two methods available in reporting skin/rib maximal dose in HDR brachytherapy treatment planning: manual selection method and planner‐independent objective method using volumetric information such as a dose‐volume histogram (DVH).^(15)^ Depending upon a dose point manually selected, the manual selection method showed interuser and intrauser variability in reporting maximal skin/rib dose. Therefore, in this study, planner‐independent objective method was used to eliminate the dose variation resulting from manual selection method. A virtual skin volume was produced by excluding volume of tissue from the expansion of skin surface external to the body with a certain thickness (i.e., 0.5 cm in Fig. 1). It was visually verified in the TPS that the virtual skin volume included the skin surface. Therefore, a skin maximal dose point was located at the skin surface the same as in the manual selection method. A skin Dmax was objectively extracted from the calculated DVH in the TPS. The rib Dmax was also extracted from the DVH based on the volume of rib delineated in planning CT images. In addition, the grid size of dose calculation matrix was set to the smallest possible value of 0.1 cm in three dimensions (3D) to minimize the impact of dosimetric uncertainty from the resolution of dose matrix.

On the 3D planning CT images, the shape and location of the balloon were reviewed and the balloon diameter and balloon‐to‐skin distance were measured and recorded for quality assurance purposes. These measurements were repeatedly performed for verification on the CT images taken prior to each treatment fraction. In addition, if the longitudinal reference line (black line marked on the shaft of the applicator) deviated from the original position during CT simulation for treatment planning, the device was manually rotated back to the original position as identified by a skin marker. In the treatment room prior to each treatment delivery, the applicator rotation was verified by visual inspection of the longitudinal reference line on the device in alignment with the skin marker, making sure that there is no applicator rotation.

### B. Two representative clinical cases

A clinical case can belong to one of three categorized groups, depending upon the relative location of the balloon device to OARs such as skin and rib: 1. Both minimum balloon‐to‐skin and minimum balloon‐to‐rib distances are ≤0.7 cm (Group I); 2. Either of the distances is ≤0.7 cm (Group II); 3. Both distances are >0.7 cm (Group III). According to the NSABP B‐39/RTOG 0413 protocol guideline, the minimum balloon‐to‐skin distance of 0.7 cm can be a guideline for safe dose delivery to skin (≥145% of the prescribed dose). The same criterion of 0.7 cm can be applied to the minimum balloon‐to‐rib distance because the rib Dmax limit in the Contura registry study is the same as the skin Dmax limit in the NSABP B‐39/RTOG 0413 protocol. For Group III, the shape of dose distribution may be fairly symmetric and similar to a spherical shape because minimum balloon‐to‐skin and balloon‐to‐rib distances are long enough to ignore the dose constraints to OARs in dwell‐time optimization. In contrast, the degree of deviation from the spherical shape of dose distribution becomes most severe in Group I due to the proximity of the balloon to both skin and rib. In this study, a patient dataset was selected for Groups I and II as a representative clinical scenario. In Case A for Group I, the Contura MLB device was implanted with the minimum balloon‐to‐skin distance of 0.5 cm and the minimum balloon‐to‐rib distance of 0.2 cm. In Case B for Group II, the device was located in proximity only to the rib with the minimum balloon‐to‐rib distance of 0.3 cm. The minimum balloon‐to‐skin distance was 1 cm (>0.7 cm).

### C. Virtual simulation of device rotation

In 3D CT image‐based planning, each Contura lumen was defined by the position coordinates of applicator points identified by a planner. In general, the single central lumen is defined by two applicator points because it is a straight line along the central axis of the balloon device. To define the four outer lumens along their curvature, a comprehensive 3D review of planning CT images was necessary to accurately depict the curved outer lumens inside the balloon.

A scenario of device rotation was virtually simulated by rotating the outer lumens to a specific rotation angle. To mimic all possible rotation scenarios, a full rotation ranging over 360° was investigated from $-180^{\circ }$ to $+180^{\circ }$ in 10° increments (total of 35 rotation scenarios). The signs in front of the rotation angle represent the orientation of rotation: clockwise (positive) and counterclockwise (negative). As the device is viewed from proximal side on 3D planning CT images, conventional numbering of outer lumens is clockwise from #1 (identified by a dummy wire) to #4 on the cross‐sectional view of device (Fig. 1(a)). The outer lumens can rotate positively (+) or negatively (−) with respect to the single central lumen. A rotation matrix was mathematically calculated from the position coordinates of two applicator points along the single central lumen. This matrix was applied to every position coordinate of applicator points along each outer lumen. After the mathematical computation for virtual rotation was completed, the rotated position coordinates for each outer lumen were manually transferred to the TPS. Accordingly, a new plan was produced based on the position coordinates of rotated outer lumens. The manual transfer was reviewed and validated by comparing applicator coordinates of the rotated plan in the TPS with those mathematically computed. The manual transfer was also verified by visually inspecting the rotated applicator in the 3D planning CT images in the TPS. The total of 35 rotated plans was compared with the clinical MDML treatment plan (i.e., original MDML plan), as well as two single‐lumen plans (SDSL and MDSL). The following dosimetric parameters were used for plan comparison analysis: PTV_EVAL (D95 and D90), skin Dmax, rib Dmax, normal breast tissue V150 and V200.

### D. Mathematically calculated rotation matrix with respect to the single central lumen

**Figure 2:**
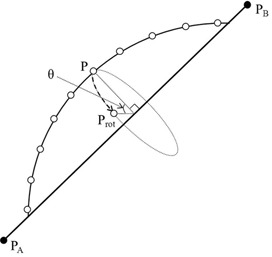
A schematic diagram shows a counterclockwise rotation from a point P(x,y,z) to a point $P_{\text{rot}}(x_{\text{rot}},y_{\text{rot}},z_{\text{rot}})$ by an angle of 9 with respect to the central lumen.

If a point P(x,y,z) is rotated to a point $P_{\text{rot}}$ ($P_{\text{rot}}(x_{\text{rot}},y_{\text{rot}},z_{\text{rot}})$ with respect to the axis of rotation defined by two points of $P_{A}\;(a,b,c)$ and $P_{B}\;(d,e,f)$ as shown in Fig. 2, the vector of rotation axis is given as follows

(1) $$\vec{P_{A}P_{B}}=(u,v,w)=(d-a,e-b,f-c)$$

with magnitude of $L=\sqrt{u^{2}+v^{2}+w^{2}}$. The rotation matrix **R** (matrix is represented in bold font) for rotation angle θ with respect to the rotation axis $\vec{P_{A}\;P_{B}}$ is mathematically calculated by the following products. The final rotated coordinate becomes $\left(x_{\text{rot}},y_{\text{rot}},z_{\text{rot}}\right)$:

(2) $$R=T_{P_{A}}^{-1}R_{\text{xz}}^{-1}R_{\text{xz}2z}^{-1}R_{z}(\theta )R_{\text{xz}2z}R_{\text{xz}}T_{P_{A}}=(x_{\text{rot}},y_{\text{rot}},z_{\text{rot}})$$

in which $T_{P_{A}}$ is the translation matrix to move starting point $\left(P_{A}\right)$ of rotating vector $\left(\vec{P_{A}P_{B}}\right)$ to the origin, $R_{xz}$ is the rotation matrix to rotate the rotating vector $\left(\vec{P_{A}P_{B}}\right)$ with respect to the z‐axis to the xz‐plane, $R_{xz2z}$ is the rotation matrix to rotate the rotating vector $\left(\vec{P_{A}P_{B}}\right)$ in the xz‐plane to the z‐axis, and $R_{z}(\theta )$ is the rotation matrix to rotate a point (P) with respect to the z‐axis by rotation angle θ where

$$\begin{array}{l} x_{\text{rot}}=\frac{a(v^{2}+w^{2})+u(-\text{bv}-\text{cw}+m)\left(\left(x-a)(v^{2}+w^{2})+u(\text{bv}+\text{cw}-\text{vy}-\text{wz}\right)\right)\text{cos}\theta +L(\text{bw}-\text{cv}-\text{wy}+\text{vz})\text{sin}\theta }{L^{2}}\\ y_{\text{rot}}=\frac{b(u^{2}+w^{2})+v(-\text{au}-\text{cw}+m)\left(\left(y-b)(u^{2}+w^{2})+v(\text{au}+\text{cw}-\text{ux}-\text{wz}\right)\right)\text{cos}\theta +L(-\text{aw}+\text{cu}+\text{wx}-\text{uz})\text{sin}\theta }{L^{2}}\\ z_{\text{rot}}=\frac{c(u^{2}+v^{2})+w(-\text{au}-\text{bv}+m)\left(\left(z-c)(u^{2}+v^{2})+w(\text{au}+\text{bv}-\text{ux}-\text{vy}\right)\right)\text{cos}\theta +L(\text{av}-\text{bu}-\text{vx}+\text{uy})\text{sin}\theta }{L^{2}} \end{array}$$

with m=ux+vy+wz

This algorithm transforms a rotation with respect to an arbitrary axis (rotating vector) defined by two points ($P_{A}$ and $P_{B}$) into a rotation with respect to the z‐axis. In addition, the starting point for the rotating vector is shifted to the origin. Therefore, if the rotation axis is parallel to the z‐axis (i.e., a=d and b=e in Eq. (1)), the products to rotate the rotation vector $\left(\vec{P_{A}P_{B}}\right)$ to the z‐axis are removed, and Eq. (2) becomes as follows:

(3) $$R=T_{P_{A}}^{-1}R_{z}(\theta )T_{P_{A}}=(x_{\text{rot}},y_{\text{rot}},z_{\text{rot}})$$

Note that in Eq. (2) the points P(x,y,z) and $P_{\text{rot}}(x_{\text{rot}},y_{\text{rot}},z_{\text{rot}})$ are mathematically represented in four homogeneous coordinates such as P (x, y, z, 1) and $P_{\text{rot}}(x_{\text{rot}},y_{\text{rot}},z_{\text{rot}},1)$ even though these points are defined in 3D space.^(16)^ This enables to do coordinate transformation (i.e., translation matrix). Hence, all matrices in Eq. (2) are four‐by‐four matrices.

## III. RESULTS

### A. MDML plan comparison with single‐lumen plans

The original MDML plan was compared with two single‐lumen plans. The dosimetric data for Case A are summarized in Table 1. PTV_EVAL coverage was similar such that D95 value (MDML plan vs. single‐lumen plans) was 98.5% vs. 98.7% of the prescribed dose and D90 value was 103.3 vs. 104.3% of the prescribed dose. MDML plan was superior to single‐lumen plans in sparing OARs. The average reduction of skin and rib Dmax values was 6.5% and 16.1% of the prescribed dose, respectively, and normal breast tissue V150 and V200 values were also reduced, on average, by 3.9 cc and 2.2 cc, respectively. All dosimetric requirements of the Contura protocol were satisfied in the MDML plan. However, skin Dmax, rib Dmax and normal breast tissue V200 values were violated in SDSL plan. Rib Dmax value was violated in MDSL plan. The asymmetric (ellipsoidal shape) dose distribution was displayed with two active (green color) lumens, as well as three inactive (pink color) lumens in Fig. 1(a) and their position changed in Fig. 1(b) due to $+70^{\circ }$ device rotation. This rotation made the positions of lumens #1 and #3 become proximal to skin and rib, as shown in Fig. 1(b) and thereby increased skin Dmax slightly from 110.6% to 118.7% of the prescribed dose. Rib Dmax value was highly elevated from 138.6% to 158.0% of the prescribed dose. The PTV_EVAL D95 and D90 values were decreased from 98.5% to 93.2% and from 103.3% to 98.4% of the prescribed dose, respectively. The change of normal breast tissue V150 and V200 values were <1 cc.

**Figure 3:**
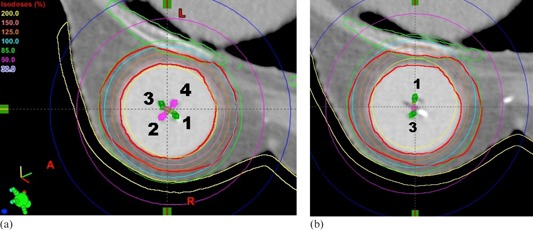
Comparison of isodose distribution between the original MDML plan (a) and a rotated plan (b) with 110° counterclockwise rotation for Case B.

The plan comparison dosimetric data for Case B are summarized in Table 2. Similar to Case A, MDML plan decreased skin and rib Dmax values, on average, by 7.6% and 28.4% of the prescribed dose, respectively. PTV_EVAL coverage was the same for D95 value (99.3% of the prescribed dose) and PTV_EVAL D90 value was 103.6 vs. 104.4% of the prescribed dose (MDML plan vs. single‐lumen plans). On average, normal breast tissue V150 was decreased by 1.6 cc, while V200 was increased by 0.8 cc. All dosimetric requirements of the Contura protocol were satisfied in MDML plan, while rib Dmax value was violated in SDSL and MDSL plans. Figure 3 shows an example of $-110^{\circ }$ device rotation for Case B. This rotation made two active lumens #1 and #3 (green color in Fig. 3(a)) close to skin and rib, as shown in Fig. 3(b), thereby tremendously increasing rib Dmax from 142.6% to 197.7% of the prescribed dose. PTV_EVAL D95 and D90 values were decreased from 99.3% to 95.5% and from 103.6% to 101.4%, respectively. The skin Dmax value was decreased from 92.4% to 85.0% of the prescribed dose and normal breast tissue V200 value was increased by 2.4 cc. The change of normal breast tissue V150 value was <0.5 cc.

**Table 1:** Dosimetric comparison of three plans (MDML, SDSL, and MDSL plans) for Case A and summary of dosimetric variations from the original MDML plan due to Contura MLB device rotation. Positive (+) and negative (−) signs in front of the rotation angle indicate the orientation of rotation such as clockwise and counterclockwise rotation, respectively

	*PTV EVAL*		*Skin*	*Rib*	*Normal Breast Tissue*	
	*D95 (%)*	*D90 (%)*	*Dmax (%)*	*Dmax (%)*	*V150 (cc)*	*V200 (cc)*
SDSL	98.6	104.8	130.6	159.5	34.6	10.8
MDSL	98.8	103.9	123.6	149.9	32.7	9.5
MDML without rotation	98.5	103.3	110.6	138.6	29.8	8.0
Minimum (Rotation angle)	$92.9(-120^{\circ })$	$98.1(-120^{\circ })$	$108.6(+150^{\circ })$	$127.5(-30^{\circ })$	$29.4(+60^{\circ })$	$7.9(-20^{\circ })$
Maximum (Rotation angle)	$99.3(-20^{\circ })$	$103.7(-20^{\circ })$	$129.0(-110^{\circ })$	$161.4(+60^{\circ })$	$29.8(+170^{\circ })$	$8.7(+70^{\circ })$
$-30^{\circ }$ Rotation	99.1	103.4	111.7	127.5	29.7	7.9
30° Rotation	95.7	100.8	115.0	152.9	29.6	8.3

a SDSL=single−dwell position for a single central lumen; MDSL=multiple−dwell positions for a single central lumen; MDML=multiple−dwell positions for multiple lumens; PTV_EVAL=planning target volume for evaluation; D95 (%) and D90 (%)=dose in percent relative to the prescribed dose enclosing 95% and 90% of PTV_EVAL volume; Dmax (%)=maximal dose in percent relative to the prescribed dose; V150 (cc) and V200 (cc)=absolute volume of organ receiving 150% and V200% of the prescribed dose.

**Table 2:** Dosimetric comparison of three plans (MDML, SDSL, and MDSL plans) for Case B and summary of dosimetric variations from original MDML plan due to Contura MLB device rotation. Positive (+) and negative (−) signs in front of the rotation angle indicate the orientation of rotation such as clockwise and counterclockwise rotation, respectively

	*PTV EVAL*		*Skin*	*Rib*	*Normal Breast Tissue*	
	*D95 (%)*	*D90 (%)*	*Dmax (%)*	*Dmax (%)*	*V150 (cc)*	*V200 (cc)*
SDSL	99.4	105.0	101.9	174.5	33.8	5.5
MDSL	99.2	103.8	97.9	167.6	31.3	4.3
MDML without rotation	99.3	103.6	92.4	142.6	31.0	5.7
Minimum (Rotation angle)	$92.8(+170^{\circ })$	$99.5(+90^{\circ })$	$84.6(-60^{\circ })$	$137.9(+110^{\circ })$	$30.8(-110^{\circ })$	$5.6(+10^{\circ })$
Maximum (Rotation angle)	$99.5(-10^{\circ })$	$104.3(-30^{\circ })$	$109.9(+80^{\circ })$	$197.7(-110^{\circ })$	$31.1(+10^{\circ })$	$8.2(-150^{\circ })$
−30% Rotation	99.4	104.3	85.9	152.5	31.0	6.3
30° Rotation	97.4	102.0	100.0	140.3	31.0	5.7

a SDSL=single−dwell position for a single central lumen; MDSL=multiple−dwell positions for a single central lumen; MDML=multiple−dwell positions for multiple lumens; PTV_EVAL=planning target volume for evaluation; D95 (%) and D90 (%)=dose in percent relative to the prescribed dose enclosing 95% and 90% of PTV_EVAL volume; Dmax (%)=maximal dose in percent relative to the prescribed dose; V150 (cc) and V200 (cc)=absolute volume of organ receiving 150% and V200% of the prescribed dose.

### B. Dosimetric variations due to device rotation

#### B.1 Case A

**Figure 4:**
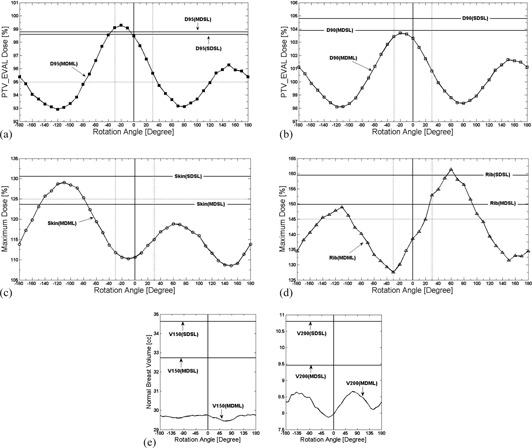
(a) PTV_EVAL coverage (D95), (b) PTV_EVAL coverage (D90), (c) skin maximal dose (Dmax), (d) rib Dmax, and (e) normal breast tissue (V150 and V200) variations due to rotation of Contura MLB applicator for two single‐lumen plans (SDSL and MDSL) and a MDML plan for Case A. Two dotted vertical lines represent $\pm 30^{\circ }$ device rotation for ((a)‐(d)). Parallel dotted line represents the minimum Contura dosimetric goal for PTV_EVAL D95 value (95% of the prescribed dose) in (a), skin Dmax value (125% of the prescribed dose) in (c), and rib Dmax value (145% of the prescribed dose) in (d), respectively.

Dosimetric variations for Case A are depicted in Fig. 4 and summarized in Table 1. The variations of D95 (Fig. 4(a)) and D90 (Fig. 4(b)) values from the original MDML plan (98.5% and 103.3% of the prescribed dose) ranged from −5.6% to 0.8% of the prescribed dose and from −5.2% to 0.4% of the prescribed dose, respectively. Because of device rotation, the D95 value of MDML plan deteriorated less than that of single‐lumen plans, except for rotation angles between 0° and ‐30° (later denoted as [$0^{\circ },-30^{\circ }$]) in which it was slightly improved (<1%). Furthermore, for rotation angles $\left[-170^{\circ },-80^{\circ }\right]$ and $\left[+40^{\circ },+120^{\circ }\right]$, it was lower than the Contura dosimetric goal.

Skin Dmax (Fig. 4(c)) varied from the original MDML plan (110.6% of the prescribed dose), ranging from −2.0% to 18.4% of the prescribed dose. The skin Dmax was increased in most rotation scenarios except for a rotation angle of $-10^{\circ }$ and angles $\left[+130^{\circ },+160^{\circ }\right]$. For rotation angles $\left[-140^{\circ },-80^{\circ }\right]$, the skin Dmax of MDML plan was higher than that of MDSL plan and violated the Contura dosimetric goal. The rib Dmax (Fig. 4(d)) variation from the original MDML plan (138.6% of the prescribed dose) widely fluctuated between −11.1% and 22.8% of the prescribed dose (range of 33.9% of the prescribed dose). The rib Dmax increased for 20 rotation scenarios, while decreasing for the remaining 15 scenarios. For rotation angles $\left[+30^{\circ },+90^{\circ }\right]$, the rib Dmax of MDML plan was higher than that of MDSL plan. Moreover, at a rotation angle of $+60^{\circ }$, it was higher than that of SDSL plan. For rotation angles $\left[-140^{\circ },-100^{\circ }\right]$ and $\left[+30^{\circ },+100^{\circ }\right]$, the rib Dmax violated the Contura dosimetric goal. The change of normal breast tissue volume V150 and V200 values (Fig. 4(e)) was small: <1 cc (0.4 cc for V150 and 0.8 cc for V200 values, respectively). V150 and V200 values of MDML plan after device rotation were still better than those of the single‐lumen plans and satisfied the Contura dosimetric goal.

Device rotation of $-30^{\circ }$ increased PTV_EVAL coverage by 0.6% of the prescribed dose for D95 value and 0.1% of the prescribed dose for D90 value, respectively. It reduced rib Dmax by 11. 1% of the prescribed dose, while slightly increasing skin Dmax by 1.1% of the prescribed dose. Both normal breast tissue V150 and V200 values were decreased by 0.1 cc. Therefore, $-30^{\circ }$ device rotation had a beneficial impact on plan dosimetry by increasing PTV_EVAL coverage and decreasing rib Dmax. In contrast, $+30^{\circ }$ device rotation caused a significant deterioration in plan dosimetry. PTV_EVAL D95 and D90 values were reduced by 2.8% and 2.5% of the prescribed dose, respectively. Normal breast tissue V150 and V200 values were slightly changed by −0.2 cc and 0.3 cc, respectively. While skin Dmax was increased by 4.4% of the prescribed dose, rib Dmax was drastically increased by 14.3% of the prescribed dose to 152.9% of the prescribed dose, which violated the Contura dosimetric goal.

#### B.2 Case B

**Figure 5:**
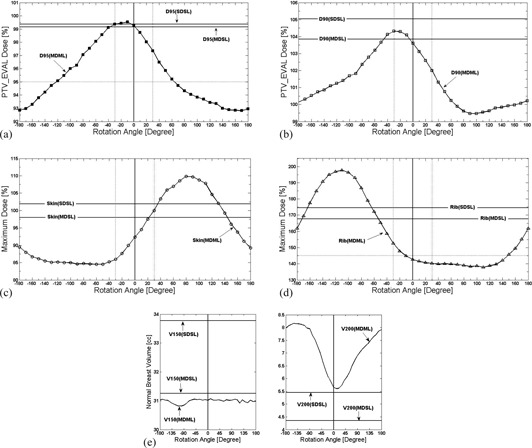
(a) PTV_EVAL coverage (D95), (b) PTV_EVAL coverage (D90), (c) skin maximal dose (Dmax), (d) rib Dmax, and (e) normal breast tissue (V150 and V200) variations due to rotation of Contura MLB applicator for two single‐lumen plans (SDSL and MDSL) and a MDML plan for Case B. Two dotted vertical lines represent $\pm 30^{\circ }$ device rotation for ((a)‐(d)). Parallel dotted line represents the minimum Contura dosimetric goal for PTV_EVAL D95 value (95% of the prescribed dose) in (a), rib Dmax value (145% of the prescribed dose) in (d), respectively.

Dosimetric variations for Case B are shown in Fig. 5 and summarized in Table 2. The variation of PTV_EVAL D95 (Fig. 5(a)) and D90 (Fig. 5(b)) values from the original MDML plan (99.3% and 103.6% of the prescribed dose) ranged from −6.5% to 0.2% of the prescribed dose and from −4.1% to 0.7% of the prescribed dose, respectively. In general, device rotation decreased the D95 value of MDML plan less than that of single‐lumen plans except for rotation angles $\left[0^{\circ },-30^{\circ }\right]$. For rotation angles $\left[-180^{\circ },-130^{\circ }\right]$ and $\left[+70^{\circ },+180^{\circ }\right]$, the D95 value was lower than the Contura dosimetric goal.

Skin Dmax (Fig. 5(c)) variation from the original MDML plan (92.4% of the prescribed dose) ranged from −7.8% and 17.5% of the prescribed dose. While decreasing in all counterclockwise (−) rotation scenarios, the skin Dmax increased in clockwise (+) rotation scenarios except for rotation angles of $\geq +170^{\circ }$. The skin Dmax of MDML plan was higher than that of MDSL plan for rotation angles $\left[+30^{\circ },+140^{\circ }\right]$ and higher than that of SDSL plan for rotation angles $\left[+40^{\circ },+130^{\circ }\right]$, respectively. Because minimum balloon‐to‐skin distance was large enough (1 cm), skin Dmax value (109.9% of the prescribed dose) was still lower than Contura dosimetric goal, even for the worst case scenario at the rotation angle of $+80^{\circ }$. On the contrary, because of the short minimum balloon‐to‐rib distance (0.3 cm), rib Dmax (Fig. 5(d)) variation from the original MDML plan (142.6% of the prescribed dose) was as high as 59.8% of the prescribed dose (ranging from −4.7% to 55.1%). Even though the rib Dmax was still under the Contura dosimetric limit for rotation angles $\left[-10^{\circ },+140^{\circ }\right]$ (range of 150°), it was higher than the limit for the remainder of rotation angles (range of 200°). In particular, the rib Dmax was higher than that of MDSL plan for rotation angles $\left[-170^{\circ },-60^{\circ }\right]$ and higher than that of SDSL plan for rotation angles $\left[-160^{\circ },-70^{\circ }\right]$. Normal breast tissue (Fig. 5 (e)) V150 variation ranged from −0.2 cc to 0.1 cc and V200 variation ranged from −0.1 cc to 2.5 cc. After rotation, the V150 value of MDML plan remained lower than that of single‐lumen plans and the Contura dosimetric limit. Though V200 value of MDML plan was much higher than that of single‐lumen plans, it was still lower than Contura dosimetric limit.

Device rotation of $-30^{\circ }$ changed plan dosimetry as follows: slight improvement of PTV_EVAL D95 and D90 values by 0.1% and 0.7% of the prescribed dose, respectively; better sparing of skin (decrease of skin Dmax by 6.5% of the prescribed dose); more dose to rib (increase of rib Dmax by 9.9% of the prescribed dose); and invariant normal breast tissue V150 value and 0.6 cc increase of V200 value. The $-30^{\circ }$ rotated plan was clinically unacceptable because rib Dmax (152.5% of the prescribed dose) violated the Contura dosimetric goal. On the other hand, $+30^{\circ }$ device rotation decreased PTV_EVAL D95 and D90 values by 1.9% and 1.6% of the prescribed dose, respectively. It increased skin Dmax value by 7.6% of the prescribed dose, while decreasing rib Dmax value by 2.3% of the prescribed dose. Normal breast tissue V150 and V200 values were invariant. Despite lower target coverage than single‐lumen plans and increased skin Dmax value, $+30^{\circ }$ rotated plans were clinically acceptable by fully satisfying Contura dosimetric goals.

## IV. DISCUSSION

The sinusoidal dose variation graphs (Figs. 4 and 5) demonstrated that device rotation could improve or deteriorate a specific dosimetry data point of the original MDML plan, depending upon rotation angle. For the representative clinical cases in this study, a certain dosimetric datum of a rotated MDML plan was inferior to that of single‐lumen plans, even for a small device rotation such as $\pm 30^{\circ }$. Furthermore, the Contura study dosimetric goal was not met in some rotated MDML plans, while it was fully met in the original MDML plan. Therefore, the verification and correction of device rotation is essential prior to delivery of each fraction.

It is noted that in most clinical cases the rotation error occurs within ± 10°. Even for two highly asymmetric dose distributions in this study, the dosimetric change due to device rotation by $\pm 10^{\circ }$ was small: < 1% of the prescribed dose for PTV_EVAL D95 and D90 values; <2.5% of the prescribed dose for skin and rib Dmax values; and <0.3 cc of normal breast tissue V150 and V200 values.

The analysis of sinusoidal dose variation graphs can reveal two additional findings. First, dose variation is highly dependent upon the magnitude of asymmetry of dose distribution which is strongly related to the proximity of OARs to the device. The more proximal to the OARs, the more asymmetric dose distribution is necessary, thereby resulting in more significant dosimetric variations due to device rotation. For both Case A and B, the device was located more proximally to rib than skin. Therefore, the rib Dmax value had more dose variation than the skin Dmax value (33.9% vs. 20.4% of the prescribed dose for Case A; 59.8% vs. 25.3% of the prescribed dose for Case B). Secondly, the sinusoidal dose variation graph could elucidate a trend of dose variation for skin and rib Dmax values, depending upon their relative location with respect to the device. If both skin and rib are located close to the device (Case A), their relative positions to the balloon are generally in opposition to each other. Along the direction of skin and rib (i.e., AP direction in Fig. 1(a)), dose distribution should be contracted to reduce maximal skin and rib dose. Device rotation which moves active catheter #1 close to skin makes active catheter #3 rotate closer to rib to the same extent. Therefore, the phase of sinusoidal dose variation graph for skin Dmax coincides with that for rib Dmax, as in Figs. 4(c) and 4(d). As rib Dmax is increased (decreased), skin Dmax is also increased (decreased). In contrast, if either skin or rib is exclusively located proximal to the device (Case B), an optimized dose distribution is skewed in Fig. 3(a) opposite to the proximal structure to spare that structure, but not both. Hence, the phase of either Dmax sinusoidal dose variation graph is inverted each other, as in Figs. 5(c) and 5(d). As rib Dmax is increased (decreased) due to device rotation, skin Dmax is decreased (increased) accordingly.

The goal of inverse planning for HDR brachytherapy is to optimize dwell‐time distribution to obtain an optimal dose distribution exactly conforming to the target volume with as low as possible dose to OARs.^(17)^ However, for a case in which OARs are located very close to the target volume, it is difficult to find an optimal set of dosimetric constraints in the inverse planning procedure to meet all dosimetric goals. In this case, it is necessary to compromise between target coverage and OARs dose sparing. For example, if more conservative planning goals are used for the inverse planning procedure, the resultant dose distribution can be a just good enough clinically (i.e., not highly asymmetric dose distribution). For this plan, the dosimetric variation due to device rotation is not severe because dose distribution is slightly asymmetric. In contrast, if very strict planning goals are used for the inverse planning procedure, the resultant dose distribution must be highly asymmetric and thereby dose variation due to device rotation should be significant. Nevertheless, dose variation due to device rotation is highly dependent upon the degree of asymmetry of resultant dose distribution from the inverse planning procedure.

Though several approaches^18^, ^19^, ^20^, ^21^ were discussed in the literature to account for tissue inhomogeneity in dose calculation for intracavity breast HDR brachytherapy, the commercial TPS in this study simply used The American Association of Physicists in Medicine (AAPM) task group (TG) 43 formalism^(22)^ without taking tissue inhomogeneity into account. In reality, path length and material composition of medium from the ${}^{192}\text{Ir}$ source at each active dwell position to a certain dose point, such as maximal skin/rib dose point, vary as the applicator rotates. If highly accurate calculation of the skin/rib maximal dose is needed, it is better to use the dose calculation algorithm that takes tissue inhomogeneity into account.

Though applicator rotation tool is available in the commercial TPS, it requires that a planner manually define rotation axis as well as rotation angle. Hence, if this manual tool is used for this device rotation study, it is difficult to eliminate dose variation resulting from human error in manual device rotation for each scenario. If the rotation angle increment is large, such as every 10° as this study, human error from the use of manual rotation tool may not be an issue because the human error would be relatively much less than 10° rotation increment. However, it will result in systematic error to manually position rotation axis and angle for small increment of rotation angle such as every 2°. Consequently, as far as device rotation study is concerned, the method using mathematically computed rotation matrix is more accurate than that using manual rotation tool in the TPS.

In the current clinical practice, applicator rotation is always verified prior to each fraction and correction is made by rotating the device back to its original position. Another remedy to correct device rotation is replanning based upon the rotated applicator geometry. Using verification images, the rotation angle can be accurately calculated and applied to applicator coordinates. Upon the rotated device coordinates, a new dwell‐time distribution must be optimized and transferred to HDR afterloader for every fraction. Alternative option for replanning is to use the verification 3D CT images as planning CT data and replanning is performed from scratch prior to every fraction: contouring target and OARs; defining dwell positions; optimizing dwell‐time distribution. Because plan optimization mainly depends on applicator geometry, both replanning techniques produce different plans from the original plan despite the same optimization parameters (dose constraints) employed in the inverse planning procedure. For instance, in Case A, original optimized MDML plan uses outer lumens #1 and #3 (Fig. 1(a)) exclusively or has higher weighting of dwell times to outer lumens #1 and #3. Because outer lumens #2 and #4 are closely located to rib and skin, respectively in Fig. 1(a), they are not used or have extremely lower weighting for optimization. On the other hand, after $+70^{\circ }$ rotation (Fig. 1(b)), reoptimized MDML plan based on the rotated applicator geometry will use outer lumens #2 and #4 exclusively or has higher weighting of dwell times to outer lumens #2 and #4 because lumens #1 and #3 are proximally located to skin and rib, respectively. However, both replanning approaches prior to every fraction are expected to tremendously increase replanning time and effort, and seem to be impractical in a routine clinical workflow.

Though this study evaluated the dosimetric impact of device rotation for a limited number of patients, the method and concept presented here can be applicable to any patient in the clinic; therefore, a larger sample size is irrelevant. A statistically strong relationship between device rotation and its dosimetric impact will be revealed if a large number of patient cohorts are recruited. Nonetheless, the dose variation is highly related to the clinical treatment plan quality (i.e., shape of dose distribution) produced for a specific patient. For a large group of patients whose dose distribution is slightly asymmetric, the dose variation due to device rotation may be clinically insignificant. For another group of patients whose balloons are located very close to both skin and rib, highly asymmetric dose distributions are necessary. Based on these data, one can ascertain that there is statistical significance of dosimetric impact due to device rotation. We believe that asymmetric dosimetric distributions for Case A and B in this study are good clinical representations.

For a future study, a computerized module or tool will be developed to automate the virtual simulation of rotation and employed to determine the optimal angle of rotation for the routine clinical use. The multilumen intracavitary balloon applicator has to be rotated to the optimal angle to result in the best treatment plan (maximizing the target coverage while minimizing the dose to OARs). Apparently, the optimal angle of device should be verified prior to each fraction and also maintained during the delivery of each fraction.

## V. CONCLUSIONS

Virtual simulation for the rotation of the Contura MLB applicator using a rotational matrix along the single central lumen could be applicable to any patient in the clinic. The resultant sinusoidal dose variation graph demonstrates that rotation of the Contura MLB applicator could negate the benefit of improved dose shaping capability from four additional offset outer lumens and make dosimetric data worse than single‐lumen plans, even with device rotation as little as 30°. Therefore, verification and correction of device rotation is essential prior to delivery of each fraction.

## ACKNOWLEDGMENTS

Authors want to appreciate James D. Christensen, Ph.D., in Allegheny General Hospital, Pittsburgh, Pennsylvania, for his effort in the preparation of some figures.

## Supporting information

Supplementary Material

Supplementary Material

Supplementary Material

Supplementary Material
