# Supplementary figures and images for: Dosimetric evaluation of multilumen intracavitary balloon applicator rotation in high‐dose‐rate brachytherapy for breast cancer
Source: J Appl Clin Med Phys. 2014 Jan 6;15(1):76–89. doi: 10.1120/jacmp.v15i1.4429 (PMC5711249; doi:10.1120/jacmp.v15i1.4429)

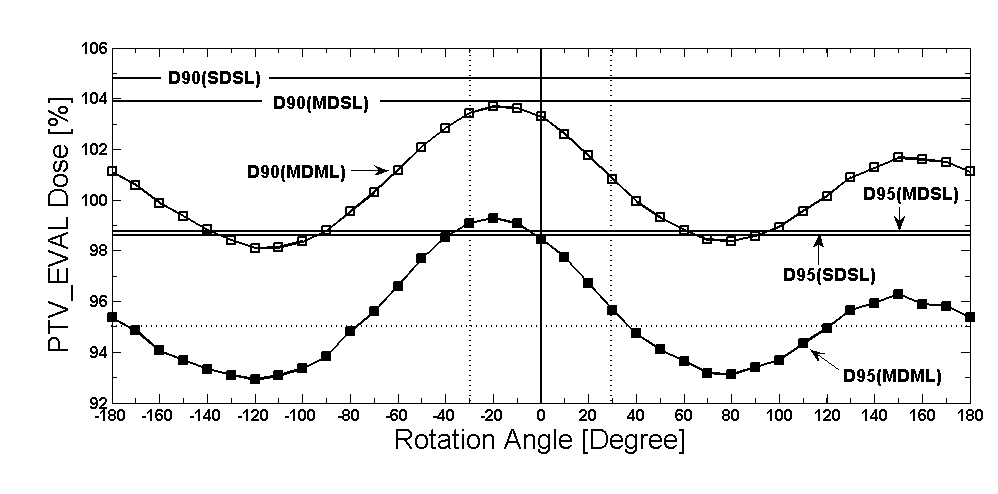

Supplement: Supplementary file 1 — Supplementary Material [file ACM2-15-076-s001.jpg]

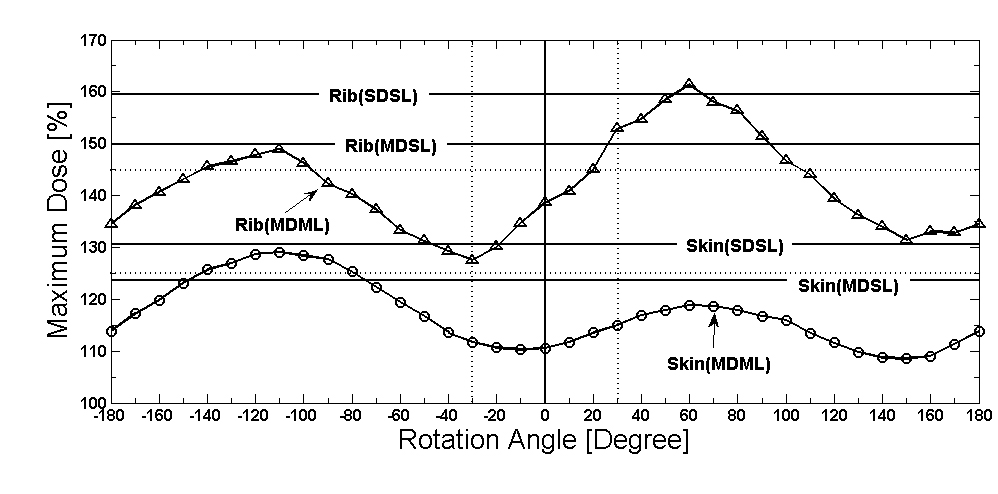

Supplement: Supplementary file 2 — Supplementary Material [file ACM2-15-076-s002.jpg]

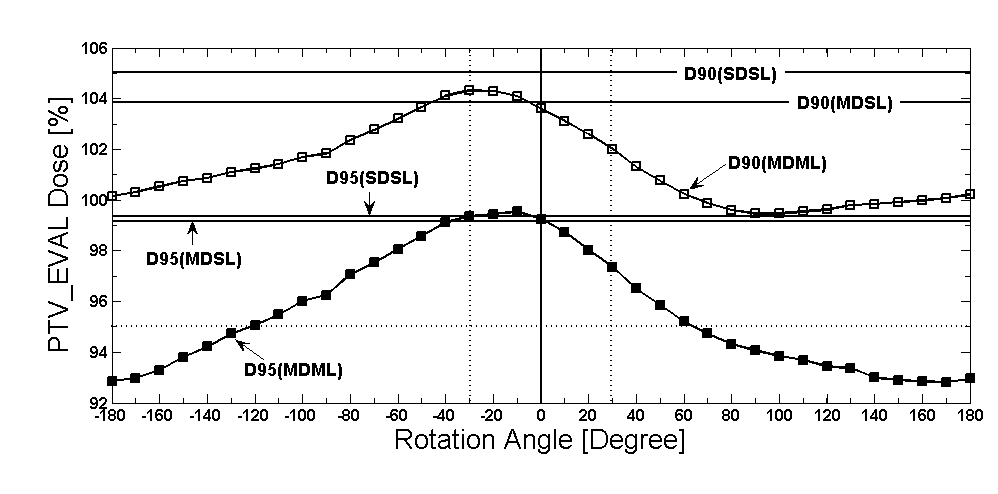

Supplement: Supplementary file 3 — Supplementary Material [file ACM2-15-076-s003.jpg]

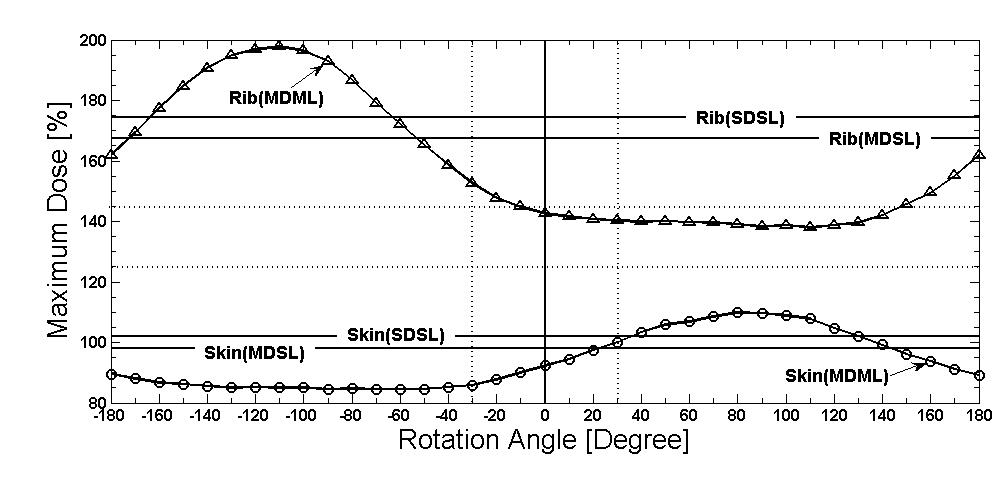

Supplement: Supplementary file 4 — Supplementary Material [file ACM2-15-076-s004.jpg]
